# The Response of CD1d-Restricted Invariant NKT Cells to Microbial Pathogens and Their Products

**DOI:** 10.3389/fimmu.2015.00226

**Published:** 2015-05-13

**Authors:** Luc Van Kaer, Vrajesh V. Parekh, Lan Wu

**Affiliations:** ^1^Department of Pathology, Microbiology and Immunology, Vanderbilt University School of Medicine, Nashville, TN, USA

**Keywords:** invariant natural killer T cells, CD1d, glycolipid antigens, microbial pathogens, microbial products, immunological unresponsiveness, immunotherapy

## Abstract

Invariant natural killer T (iNKT) cells become activated during a wide variety of infections. This includes organisms lacking cognate CD1d-binding glycolipid antigens recognized by the semi-invariant T cell receptor of iNKT cells. Additional studies have shown that iNKT cells also become activated *in vivo* in response to microbial products such as bacterial lipopolysaccharide, a potent inducer of cytokine production in antigen-presenting cells (APCs). Other studies have shown that iNKT cells are highly responsive to stimulation by cytokines such as interleukin-12. These findings have led to the concept that microbial pathogens can activate iNKT cells either directly via glycolipids or indirectly by inducing cytokine production in APCs. iNKT cells activated in this manner produce multiple cytokines that can influence the outcome of infection, usually in favor of the host, although potent iNKT cell activation may contribute to an uncontrolled cytokine storm and sepsis. One aspect of the response of iNKT cells to microbial pathogens is that it is short-lived and followed by an extended time period of unresponsiveness to reactivation. This refractory period may represent a means to avoid chronic activation and cytokine production by iNKT cells, thus protecting the host against some of the negative effects of iNKT cell activation, but potentially putting the host at risk for secondary infections. These effects of microbial pathogens and their products on iNKT cells are not only important for understanding the role of these cells in immune responses against infections but also for the development of iNKT cell-based therapies.

## Introduction

The immune response to microbial pathogens is orchestrated by reciprocal interactions between various components and cells of the innate and adaptive immune systems. While cells of the innate immune system recognize foreign invaders via conserved receptors that bind molecular patterns contained within a variety of microorganisms, cells of the adaptive immune system recognize foreign invaders via highly diverse antigen receptors that exhibit substantial pathogen-specificity. A key aspect of the adaptive immune system is its capacity to remember prior encounters with the same antigen, a property that forms the basis for the efficacy of vaccines. In addition to immune cells that can be clearly labeled as belonging to the innate or adaptive arms of the immune system, studies over the past few decades have identified several lymphocyte subsets that express antigen-specific receptors, yet exhibit many characteristics typical of cells of the innate immune system. This family of cells includes both B and T lineage cells and is often referred to as innate-like B and T lymphocytes (1). Innate-like B cells include B-1a and B-1b B cells, subsets of regulatory B (B_reg_) cells, marginal zone (MZ) B cells, and innate response activator (IRA) cells. Innate-like T cells include subsets of γδ T cells, mucosal T cells expressing CD8αα homodimers, mucosal-associated invariant T (MAIT) cells, and natural killer T (NKT) cells. Each of these cell types expresses a limited repertoire of antigen-specific receptors, responds rapidly to antigenic stimulation, and is unable to induce long-lasting immunity. These cells cannot be easily categorized as innate or adaptive, and have therefore been referred to as “inbetweeners” (2). Several of these cell types reside at mucosal surfaces, body cavities, or entry points of lymphoid organs, where they are one of the first cell types to interact with pathogens, thus playing a sentinel function in the immune system. These cells, through recognition of non-specific innate immune signals and production of immunomodulatory cytokines, interact with and influence the function of multiple cell types in the innate and adaptive branches of the immune system, and thus shape subsequent inflammatory responses and impact disease outcomes. Such innate effector functions permit these cells to respond rapidly during the early stage of immune and inflammatory responses and serve as a bridge to adaptive immunity.

In this review article, we focus on NKT cells, and particularly the subset of NKT cells called invariant natural killer T (iNKT) cells. These cells play a critical role in the immune response against a variety of microbial pathogens, a topic that is described in a number of excellent review articles (3–5). Here, we focus on the mechanisms of iNKT cell activation by microbial pathogens and the dynamics of the ensuing iNKT cell response.

## General Properties and Functions of iNKT Cells

Natural killer T cells are a subset of T lymphocytes that recognize lipid and glycolipid antigens when bound with the major histocompatibility complex (MHC) class I-related protein CD1d (6–9). Because the nomenclature of NKT cells and related cell types is confusing, we refer the reader to an opinion article on this topic (10). Two subsets of NKT cells have been identified: type 1 or iNKT cells express a semi-invariant T cell receptor (TCR), whereas type 2 or variant NKT (vNKT) cells express more diverse, yet oligoclonal TCRs (10).

Murine iNKT cells express Vα14-Jα18 chains paired with either Vβ8.2, -7, or -2 chains, and human iNKT cells express homologous Vα24–Jα18 chains paired with Vβ11. These cells also express a variety of receptors such as NK1.1 (expressed in some mouse strains) and members of the Ly49 family that are characteristic of the natural killer (NK) cell lineage. iNKT cells also express surface markers such as CD25, CD44, and CD69, which are characteristic of activated and memory T cells. The majority of iNKT cells also express the co-receptor CD4, and a small subset of human (but not mouse) iNKT cells expresses CD8α. iNKT cells are most abundant in spleen, liver, thymus, and bone marrow, and are also found in lymph nodes, peripheral blood, adipose tissue, skin, and mucosal surfaces in the intestine and lungs. In humans, iNKT cells are less abundant than in mice and their prevalence varies widely among different individuals, for reasons that remain unclear.

Following their activation iNKT cells can quickly elicit an effector response, including rapid cytokine production and cytotoxicity, making them a very crucial component of the immune response (11). Activation of iNKT cells with a cognate ligand induces secretion of a wide variety of cytokines, chemokines, and colony-stimulating factors. During this activation process, iNKT cells also interact with other cells of the immune system, resulting in their activation, recruitment, and/or differentiation (12). While iNKT cells can simultaneously produce multiple cytokines, it is now clear that subsets of iNKT cells producing distinct cytokines and with distinct effector functions exist. This includes Tbet^+^ NKT1 cells producing IFN-γ, GATA3^+^ NKT2 cells producing IL-4, RORγt^+^ NKT17 cells producing IL-17A, IL-21, and IL-22 (13), and Bcl6^+^ follicular helper NKT (NKT_FH_) cells producing IL-21 (14). iNKT cells with immunosuppressive functions have also been identified, including regulatory NKT10 cells producing IL-10 (15), E4BP4^+^ regulatory iNKT cells in adipose tissue producing IL-2 and IL-10 (16), and Foxp3^+^ regulatory iNKT cells (17). Whether the latter cell types represent separate subsets of regulatory iNKT cells remains unclear (18).

Because of their ability to produce such a mixture of cytokines and to interact with a variety of other cells of the immune system, iNKT cells can either promote or suppress immune responses in different disease conditions (11, 19). They confer natural immunity to cancer (20), provide protective immunity to various infectious agents (3–5), generally play a suppressive role during autoimmune responses (18) and graft-vs.-host disease (21), and contribute to the development of allergic airway reactivity (22), contact hypersensitivity (23), experimental hepatitis (24), atherosclerosis (25), and obesity-associated insulin resistance (26). Because iNKT cells display such a wide variety of versatile functions, they have been referred to as the “Swiss army knife of the immune system” (27).

In keeping with their immunoregulatory functions, numerous studies have explored the therapeutic activities of iNKT cells in a variety of diseases (6–9, 11, 12). Many of these studies have been performed with the prototypical iNKT cell antigen α-galactosylceramide (α-GalCer), a potent iNKT cell agonist that was originally isolated from a marine sponge (28). α-GalCer and related glycolipids have potent anti-metastatic activities (29), hasten clearance of some microbial pathogens (3–5), enhance the efficacy of vaccines (30, 31), prevent graft-vs.-host disease (21), and protect against autoimmunity in experimental models for type 1 diabetes (32), multiple sclerosis (33), arthritis (34), and systemic lupus erythematosus (35).

## Mechanisms of iNKT Cell Activation by Microbial Pathogens

Invariant natural killer T cells become activated in response to challenge by a variety of microorganisms, including bacteria, viruses, fungi, and protozoa (5). While some of these microorganisms contain glycolipid or phospholipid antigens that can bind with CD1d to activate the iNKT cell TCR, most microorganisms activate iNKT cells independently of cognate antigens. iNKT cells are highly responsive to stimulation by certain types of cytokines, which may be induced in antigen-presenting cells (APCs) via engagement of pathogen recognition receptors (PRRs) with pathogen-associated molecular patterns (PAMPs). Activation of iNKT cells via superantigens has also been reported. We will briefly discuss these distinct modes of iNKT cell activation in the following sections.

### iNKT cell activation by microbial lipid antigens

**Figure 1:**
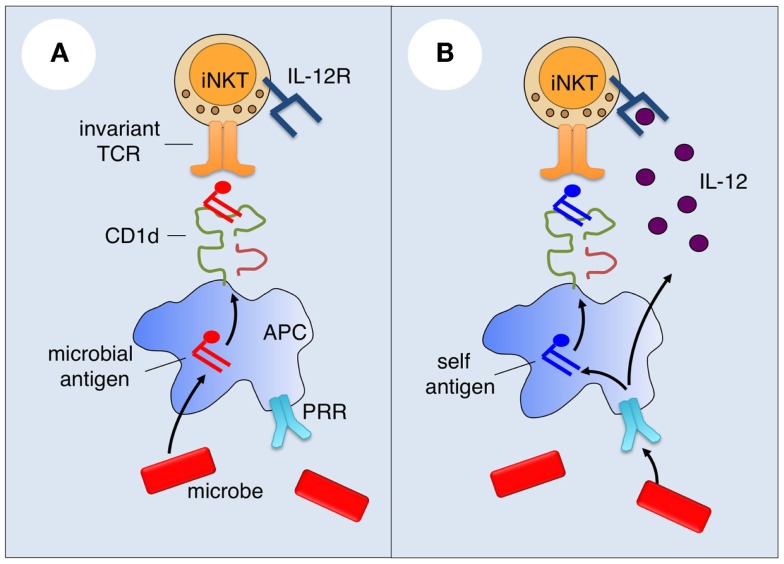
**Mechanisms of microbial iNKT cell activation**. **(A)** For microbes that contain iNKT cell antigens, such antigens may be sampled by antigen-presenting cells (APCs) and loaded onto CD1d for presentation and activation of iNKT cells. **(B)** Microbes lacking or containing iNKT cell antigens can activate iNKT cells by innate cytokine-driven mechanisms. Microbial products may engage pattern recognition receptors (PRRs) on APCs to induce cytokines such as IL-12 that bind with cytokine receptors on iNKT cells, and the production of endogenous iNKT cell antigens.

A number of microorganisms, especially bacteria, contain lipid antigens that can activate iNKT cells (Figure 1A). *Sphingomonas* species, which include organisms that are ubiquitous in the environment, produce glycosphingolipids with α-linked glucuronic or galacturonic acid (36–38), and *Borrelia burgdorferi* (39) and *Streptococcus pneumoniae* (40) contain diacylglycerols with α-linked glucosyl or galactosyl moieties that are recognized by the iNKT cell TCR. Other documented or proposed iNKT cell antigens include phosphatidylinositol mannoside from *Mycobacterium bovis* (41), a cholesterol ester with an α-linked glucoside from *Helicobacter pylori* (42), an α-GalCer from the common gut bacterium *Bacteriodes fragilis* (43), lipophosphoglycans from the protozoan parasites *Leishmania donovani* (44) and *Entamoeba histolytica* (45), and the glycosphingolipid asperamide B from the fungal pathogen *Aspergillus fumigatus* (46). While most of these antigens activate all iNKT cells, some likely activate only a subset of iNKT cells (5). Interestingly, one study showed that *B. fragilis* contains, in addition to an iNKT cell-activating α-GalCer, an inhibitory α-GalCer (Bf717) that regulates the homeostasis of host intestinal iNKT cells (47).

Some of the microbial antigens, especially those derived from *Sphingomonas* bacteria, bear structural similarity with α-GalCer, the prototypical iNKT cell antigen obtained from the marine sponge *Agelas mauritianus* (28). This finding led to speculation that α-GalCer might, in fact, be derived from bacteria, possibly *Sphingomonas* species, that colonize the sponge. As *Sphingomonas* bacteria are ubiquitous in the environment, including soil and the ocean, this is a likely yet unproven explanation for the rather strange capacity of sponge-derived products to activate a small subset of cells in the mammalian immune system.

While purified or synthetic versions of microbial antigens can potently activate iNKT cells both *in vitro* and *in vivo*, the contribution of these antigens to the response of iNKT cells to intact microorganisms is less clear. Instead, the available evidence suggests that innate cytokine-driven signals, rather than microbial antigens, are the main drivers of iNKT cell activation during microbial infection (5).

### Cytokine-driven iNKT cell activation during microbial infection

A major difference between conventional T cells and iNKT cells is that the latter but not the former are highly responsive to innate and cytokine-driven signals (5). iNKT cells constitutively express a number of cytokine receptors, most notably the receptors for IL-12 (48) and IL-18 (49). Consistent with their innate effector functions, stimulation of iNKT cells with IL-12 or IL-18 can induce IFN-γ production by these cells. In this context, iNKT cells have been shown to play a critical role in the anti-tumor activities of IL-12 (50). IL-12 has been implicated in the capacity of many microorganisms to activate iNKT cells. This phenomenon was initially described for *Salmonella typhimurium*, a Gram-negative bacterium that lacks cognate iNKT cell antigens (51). APCs cultured with this organism were able to induce IFN-γ production by iNKT cells, which was blocked by addition of neutralizing anti-IL-12 antibodies. In addition to the intact microorganisms, *S. typhimurium* lipopolysaccharide (LPS) similarly activated iNKT cells in an IL-12-dependent manner, suggesting a critical role for toll-like receptor (TLR) activation in the APCs. These findings lead to the concept that microbes lacking cognate antigens can activate iNKT cells in a manner that involves TLR signaling in APCs, production of IL-12 by the APCs, and IL-12R signaling in iNKT cells (Figure 1B). This concept has been tested and extended to iNKT cell activation by a variety of microorganisms, including viruses, bacteria, fungi, and protozoa (5). It was shown that TLR ligands for either cell surface or endosomal TLRs may be involved and, in the case of fungi, β-glucans that signal through Dectin-1 on APCs can similarly activate iNKT cells (52). While IL-12 played a critical role in iNKT cell activation induced by many microbes, IL-18 was the dominant APC-derived cytokine responsible for iNKT cell activation to LPS derived from *Escherichia coli* (53), and type 1 interferons played a dominant role in the activation of iNKT by the TLR-9 agonist CpG (54). Based on these findings, a general model has emerged for the activation of iNKT cells by microorganisms that lack cognate antigens (Figure 1B): PAMPs activate APCs (predominantly DCs) to produce pro-inflammatory cytokines, which, in turn, activate iNKT cells. As already mentioned, additional evidence suggests that this might also be the dominant pathway for iNKT cell activation by many microbes that contain iNKT cell antigens (55).

An unusual mode of cytokine-driven iNKT cell activation was observed for hepatitis B virus (HBV) (56). HBV induces secretory phospholipases in infected hepatocytes that convert phosphatidylethanolamine to lysophospholipids. The lysophospholipids bind CD1d to activate type 2 NKT (vNKT) cells that in turn induce IL-12 production by APCs to indirectly activate iNKT cells. These findings are consistent with prior studies providing evidence that activation of vNKT cells with cognate antigens can lead to the trans-activation of iNKT cells (57).

A topic of some debate is whether iNKT cells themselves express functional TLRs and, thus, might be activated directly by PAMPs independently of APCs (5, 58). One study showed that TCR engagement on iNKT cells can induce TLR expression, which was able to enhance iNKT cell activation following TLR stimulation (59). Whether direct TLR engagement on iNKT cells contributes to their activation during microbial infections remains to be explored.

During the original studies with *S. typhimurium*, it was found that iNKT cell activation in the *in vitro* cultures could be partially blocked with anti-CD1d antibodies, suggesting a role for TCR engagement on iNKT cells (51). Similar observations were made for a number of other microbes (5, 60). These findings suggested that microbes activate iNKT cells in a manner that involves both cytokine receptor- and TCR-mediated signaling (Figure 1B). However, activation of iNKT cells by some microbes such as murine cytomegalovirus (MCMV) (61) and by microbial products such as *E. coli* LPS (53), did not appear to require TCR signaling, suggesting that cytokine signaling is sufficient to activate iNKT cells during infections.

While some cytokines such as type 1 interferons can induce CD1d expression (62), microbial infection is not always associated with an increase in CD1d expression on APCs. In fact, several microbes interfere with CD1d expression, presumably in an attempt to avoid iNKT cell responses (63). Nevertheless, induction of CD1d expression on APCs might contribute to iNKT cell activation during certain infections.

An appealing hypothesis emerging from these studies was that microbes might induce endogenous lipid antigens for iNKT cells (Figure 1B). This possibility was supported by the finding that microbial products can induce enzymes involved in glycosphingolipid synthesis and that inhibitors of this pathway can suppress iNKT cell activation by certain microbial products (64). Much debate has focused on the nature of the relevant glycolipid(s) involved. While iNKT cells potently react with α-linked but not β-linked glycolipids, production of glycosphingolipids in mammals has long been assumed to be limited to β-linked anomers. Nevertheless, additional studies identified the β-linked glycosphingolipid isoglobotrihexosylceramide (iGb3) as a weak self-antigen that was also suggested to be involved in iNKT cell activation in response to microbial products (37, 65). Subsequent studies cast doubt on this possibility and instead provided evidence that β-linked glucosylceramides (β-GluCer) that accumulate in mammalian cells in response to microbial products are the relevant self-antigens that synergize with APC-derived cytokines during the activation of iNKT cells by microbial products (66). The latter studies were predominantly performed with synthetic versions of β-GluCer, which, as it turned out, contained minuscule amounts of α-anomeric GluCer (67, 68). Careful studies with iNKT cell-stimulating glycosphingolipids enriched from mammalian cells eventually led to the conclusion that mammalian cells produce small amounts of α-linked glycosphingolipids such as α-GalCers and α-GluCers that can activate iNKT cells (67, 68). However, the enzymatic pathways involved in the synthesis of these antigens remain to be identified.

Most of the studies implicating a role of CD1d and self-antigens in the capacity of microbes or their products to activate iNKT cells were performed *in vitro*. The contribution of TCR engagement to *in vivo* iNKT cell activation by microbes therefore remained unclear. Surprisingly, using a reporter mouse that can detect TCR signaling, a recent study showed that *S. typhimurium* and several TLR ligands were able to activate iNKT cells in a TCR-independent manner (69). Therefore, these findings indicate that many microbes can activate iNKT cells in the absence of TCR signaling.

### Superantigen-mediated iNKT cell activation

Superantigens are microbial toxins that cause non-specific activation of T cells by engaging MHC class II molecules and the variable region of the β-chain of the TCR. *Staphylococcal* enterotoxin B (SEB) interacts with Vβ8, which is expressed by a majority of iNKT cells. SEB was able to activate Vβ8-expressing iNKT cells in a CD1d-independent manner (70, 71).

## The *in vivo* Response of iNKT Cells to Glycolipid Antigens

**Figure 2:**
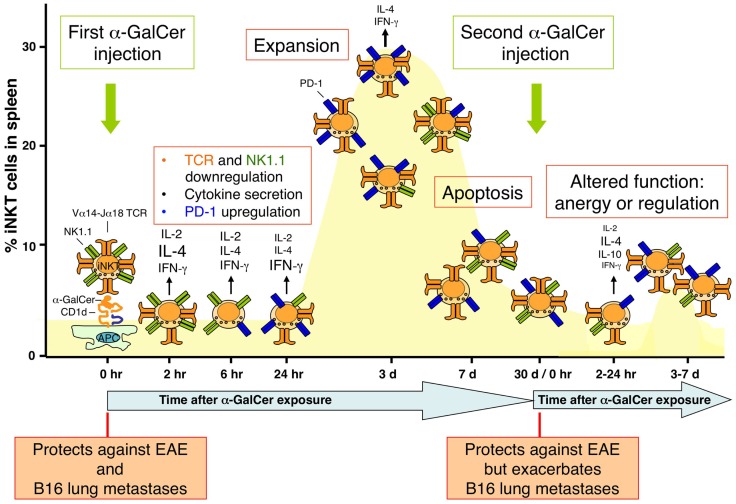
**Population dynamics of iNKT cells in response to glycolipid antigen stimulation**. The dynamics of the iNKT cell population in the spleen following intraperitoneal injection of α-GalCer in mice is shown. The percentage of iNKT cells compared with all T cells in the spleen is depicted. Injection of α-GalCer causes alterations in a variety of surface receptors on iNKT cells, including the invariant T cell receptor (TCR), NK1.1, and programmed death-1 (PD-1), as well as rapid cytokine production. The iNKT cell population rapidly expands, reaching a maximum around 3 days after α-GalCer treatment, after which most of the cells undergo apoptosis to return to homeostatic levels around 2–3 weeks. These α-GalCer-experienced iNKT cells exhibit functional defects, as revealed by their resistance to proliferate and produce large amounts of cytokines upon α-GalCer restimulation. The phenotype of these cells is reminiscent of anergic or regulatory cells. As indicated in the bottom of the figure, this altered phenotype of α-GalCer-experienced iNKT cells impacts the therapeutic activities of α-GalCer against B16 tumor metastases but not experimental autoimmune encephalomyelitis (EAE).

Most studies that have investigated the *in vivo* response of iNKT cells have focused on synthetic glycolipid antigens, most notably KRN7000, an optimized version of the original sponge-derived α-GalCer. These studies have revealed that the *in vivo* response of iNKT cells to an intraperitional injection of α-GalCer is characterized by the following series of events (Figure 2) (72):

1. Prompt activation and cytokine production: α-GalCer is presented to iNKT cells predominantly by CD8α-expressing DCs and potentially some macrophages. iNKT cell activation involves induction of a variety of activation markers (e.g., CD69, CD25, and ICOS), as well as cytokine production, with an initial burst of IL-4 (as soon as 1 h after treatment with a peak at 4 h) followed by IFN-γ (peaks at 24 h). However, this cytokine production gradually diminishes to very low levels at 3 days after treatment (11).
2. Cross-talk with other cell types: α-GalCer-activated iNKT cells engage in extensive cross-talk with other immune cell types (11). This includes activation, induction of cytokine production (most notably IL-12), and differentiation of DCs and macrophages, modulation of neutrophils, recruitment and modulation of the suppressive activities of myeloid-derived suppressor cells, profound activation and induction of IFN-γ production by NK cells, modulation of B cell and antibody responses, and modulation of CD8 and CD4 T cell responses. Most studies have provided evidence that α-GalCer treatment promotes Th2-dominant immunity. These effects form the basis of the immunomodulatory and therapeutic properties of α-GalCer and other iNKT cell antigens (12).
3. TCR downregulation: quickly following their activation by α-GalCer, iNKT cells profoundly downregulate their TCR (73). This is due to agonist-mediated inhibition of TCR recycling to the cell surface and makes these cells nearly undetectable by staining with anti-CD3 antibodies, anti-TCR antibodies, and CD1d-tetramers for a short time period, around 12–30 h after treatment.
4. NK1.1 downregulation: NK1.1 downregulation starts around 24 h after treatment, making it hard to accurately detect iNKT cells by anti-NK1.1 antibodies for an extended time period (19, 73). NK1.1 expression slowly returns to normal levels, but only about half of these cells express NK1.1 at 1 month after treatment.
5. Induction of the programmed death-1 receptor: programmed death-1 (PD-1) is an inhibitory member of the CD28 family of co-stimulatory molecules by interacting with its ligands PD-L1 and PD-L2. PD-1 has received a lot of interest in the tumor immunology field as a potent immune checkpoint whose blockade can unleash anti-tumor responses. PD-1 expression by iNKT cells is evident as early as 2–3 days after α-GalCer treatment and is sustained for up to 2 months (74–76).
6. Population expansion: iNKT cells expand in spleen and to a lesser extent in peripheral blood, bone marrow, and liver (73, 77). Expansion is maximal around 3 days after α-GalCer treatment and reaches levels about 10- to 15-fold over the starting population in spleen.
7. Apoptosis and return to homeostatic levels: following their expansion, most iNKT cells undergo apoptosis and the iNKT cell population returns to relatively normal homeostatic levels around 2–3 weeks after their expansion (73, 78). Apoptosis of iNKT cells involves the pro-apoptotic Bcl-2 family member Bim (77) and Fas/FasL interactions (79).
8. Acquisition of a hyporesponsive phenotype: as revealed by a blunted response to α-GalCer re-injection after the initial α-GalCer treatment, α-GalCer-experienced iNKT cells become unresponsive to α-GalCer restimulation (19, 78). This hyporesponsiveness was observed in terms of reduced iNKT cell activation (lack of induction of activation markers), blunted proliferation and cytokine production (IFN-γ production was more profoundly blunted than IL-4 production), and reduced capacity to activate other cell types such as DCs and NK cells. This hyporesponsive phenotype was evident between 3 days and up to 2 months after the original α-GalCer treatment. This hyporesponsive phenotype of iNKT cells was largely intrinsic to these cells and has been referred to as iNKT cell anergy. The induction and to a lesser extent the maintenance of this anergic phenotype involves PD-1/PD-L interactions (74–76, 80), as well as the egr2/3 transcription factors (80), which induce the E3 ligase Cbl-b that monoubiquitinates the CARMA1 signaling molecule in the NF-κB signaling pathway (81). An alternative explanation for the long-term effects of α-GalCer on iNKT cells proposed more recently is that the iNKT cells in α-GalCer-experienced mice adopt a regulatory phenotype with production of IL-10 (i.e., NKT10 cells) (15). Regardless of the mechanism involved, it has been established that α-GalCer-experienced iNKT cells exhibit impaired anti-tumor responses but retain their capacity to protect mice against experimental autoimmune encephalomyelitis (EAE), a mouse model of multiple sclerosis (19).

In addition to α-GalCer, the response of iNKT cells to a variety of other glycolipids has been investigated (11, 12). Many of these studies have focused on the therapeutic properties of iNKT cells and have identified glycolipid antigens that induce biased cytokine responses in iNKT cells in attempt to enhance either their anti-tumor activities or their protective effects against autoimmune or inflammatory diseases. Additional studies have explored methods to prevent or overcome the altered phenotype of α-GalCer-experienced iNKT cells. This has been accomplished by delivering α-GalCer in the context of strong co-stimulation such as α-GalCer-loaded DCs (19, 82), via intradermal, intranasal, or oral rather than systemic administration (83, 84), nanoparticles (85), recombinant CD1d molecules (86), or PD-1/PD-L blockade (74, 75, 87). Additionally, glycolipids that can potently activate iNKT cells, yet largely lack the long-term effects on iNKT cells associated with α-GalCer, have also been developed (88).

These methodologies to prevent induction of iNKT cell functional impairments are particularly important for developing improved iNKT cell-based therapies. Clinical studies with human subjects have shown long-term effects of free glycolipid treatment on human iNKT cells (89). Repeated free α-GalCer treatment resulted in increasingly weaker biological responses, which was consistent with the acquisition of iNKT cell dysfunction upon α-GalCer stimulation. Interestingly, delivery of α-GalCer in the context of DCs was able to avoid the induction of iNKT cell dysfunction in human cancer patients (90). In this context, it is also worth noting that several preclinical studies have shown that the anti-metastatic activities of α-GalCer synergize with those of PD-1/PD-L blockade (75, 87). Thus, the therapeutic activities of iNKT cells may be enhanced by methods that prevent the induction of activation-induced iNKT cell dysfunction.

## The *in vivo* Response of iNKT Cells to Microbes

Our knowledge regarding the *in vivo* response of iNKT cells to glycolipid antigens has been employed as a framework to explore the response of these cells to microbes. Most microbes and many of their products can activate iNKT cells to express a variety of activation markers and to induce cytokine production, with wide effects on other immune cell types and the outcome of the infection (5). While α-GalCer induces both IL-4 and IFN-γ production by iNKT cells, microbes typically induce little IL-4 production, which is consistent with the notion that most microbes activate iNKT cells in an innate cytokine-driven manner and that IL-12 promotes an IFN-γ-biased cytokine profile in these cells. When investigated, PD-1 upregulation was not observed, but sustained NK1.1 downregulation was common. In sharp contrast with α-GalCer, microbes or their products rarely induce systemic iNKT cell expansion *in vivo* (as discussed below, *M. bovis* is an exception), and this is true even for microbes containing iNKT cell antigens. Nevertheless, an accumulation of iNKT cells has been observed in some infected organs, such the lungs of mice infected with *Cryptococcus neoformans* (91) and the liver of mice infected with malaria parasites (92). A phenomenon observed for some microbes, including systemic infection with lymphocytic choriomeningitis virus (LCMV) (93) and *L. monocytogenes* (94, 95), is partial or complete iNKT cell depletion, which may last for several weeks.

Systemic exposure to a number of microorganisms, including *E. coli*, *S. aureus*, *S. typhimurium*, *L. monocytogenes*, and *M. bovis*, has long-term effects on iNKT cell kinetics and functions, resulting in a hyporesponsive phenotype reminiscent of that observed following α-GalCer treatment (94–96). A similar phenotype was observed for mice treated with TLR agonists such as LPS and flagellin (95, 96). Induction of this hyporesponsive phenotype required IL-12 expression (95), which itself does not induce iNKT cell dysfunction and is not required for α-GalCer-induced iNKT cell hyporesponsiveness. Furthermore, microbe-induced iNKT cell dysfunction involved both iNKT cell-intrinsic and -extrinsic mechanisms and was independent of the PD-1/PD-L pathway. While α-GalCer-experienced iNKT cells exhibited more profound defects in IFN-γ than IL-4 cytokine production, the opposite was true for the functional alterations of iNKT cells in response to microbes. Thus, the mechanisms involved in the induction of iNKT cell dysfunction mediated by glycolipids and microbes appear to be distinct. Whether specific regulatory iNKT cell subsets expand during microbial infections has not been explored. iNKT cells from mice systemically exposed to *E. coli* exhibited impairments in their therapeutic activities against metastatic tumors, but not in their capacity to protect mice against EAE (95). The latter finding might have important implications when considering iNKT cell-based therapies, as they suggest that the functions and therapeutic activities of iNKT cells in patients are influenced by recent infections.

A few studies have investigated the response of human iNKT cells to microbial pathogens. HIV infection substantially decreases iNKT cell numbers and functions, and this depletion was most profound for the CD4^+^ subset (97–99). The reduced numbers of iNKT cells may be due to a combination of HIV infection and induction of apoptosis (97, 99). Interestingly, the residual iNKT cells in infected individuals exhibited impaired ability to proliferate and produce IFN-γ in response to α-GalCer stimulation, and expressed elevated levels of PD-1 (100). Blocking experiments indicated that these functional defects were largely PD-1-independent (100). A similar although less profound reduction in iNKT cell numbers was observed in patients with active *M. tuberculosis* infection (101). The poor response of iNKT cells from these patients to α-GalCer was found to be due to increased iNKT cell apoptosis and iNKT cell dysfunction. The latter was associated with an elevated expression of PD-1, and blockade of PD-1 signaling was able to enhance the response to α-GalCer (101). iNKT cells were found to be activated during acute dengue virus infection, and the level of activation was associated with disease severity (102). These cells also exhibited reduced functional responses to subsequent α-GalCer stimulation but mechanisms were not explored (102). These studies suggest that at least some of the findings obtained in mice may also apply to infections in humans.

The response of iNKT cells to microbial pathogens makes sense from the standpoint of host–pathogen interactions. The effector functions of iNKT cells play a critical role by influencing the behavior of cells of the innate arm of the immune system and to assist in the initiation and differentiation of adaptive immune responses. Thus, iNKT cells predominantly contribute to early immune responses and their capacity to produce cytokines should therefore largely be limited to a relatively short time window early in an infection. Overactivation of iNKT cells is known to cause severe immunopathology such as liver damage (103, 104). As iNKT cells produce large bursts of cytokines with potent pro-inflammatory properties, the cytokine production potential of these cells needs to be tightly controlled to avoid the generation of a cytokine storm or a chronic inflammatory response. This may be accomplished by inducing apoptosis or functional impairments in these cells. One potential disadvantage of this strategy is that it might put the host at risk for developing secondary infections with organisms that depend on iNKT cells for protective immunity.

As responses of iNKT cells to distinct types of microbes are quite divergent, we briefly discuss below the response of iNKT cells to select microbial organisms.

### *Listeria* *monocytogenes*

Intravenous inoculation of *L. monocytogenes* resulted in rapid induction (within 1 day) of the activation marker CD69 on iNKT cells and these cells produced IFN-γ but not IL-4 (94, 95). This activation resulted in a gradual reduction in the number of iNKT cells in spleen and liver. This reduction in the prevalence of CD1d/α-GalCer-tetramer^+^ cells was not just due to activation-induced downregulation of TCR expression. These cells recovered by week 4 in the liver but not spleen. NK1.1 was downregulated for an extended time period and closely mirrored its expression following α-GalCer treatment. iNKT cells in *L. monocytogenes*-experienced mice showed reduced responses to α-GalCer in terms of CD69 induction, expansion, cytokine production (with more profound effects on IL-4 than IFN-γ), and trans-activation of DCs and NK cells. These reduced responses were not just due to lower numbers of iNKT cells but involved iNKT cell dysfunction, which was present for at least 1 month after infection. iNKT cells have been shown to play a protective role in the immune response against *L. monocytogenes* (105).

### *Mycobacterium* *bovis*

Intravenous inoculation of *M. bovis* vaccine strain bacillus Calmette–Guérin (BCG) causes an increase in CD69 expression on iNKT cells by day 7, which was further increased at day 14 in both spleen and liver (96). NK1.1 became markedly downregulated for an extended time period. Alterations in PD-1 or CTLA-4 expression by iNKT cells were not detected. Numbers of iNKT cells increased following infection, expanding approximately twofold in the spleen and fivefold in the liver at 7 days after infection. Numbers of iNKT cells subsequently contracted, reaching pre-infection levels around 2–3 weeks after infection. iNKT cell death was associated with an increase in Fas expression on these cells. iNKT cells were able to produce IFN-γ quickly following infection, reaching a peak at day 7, but the capacity to produce IFN-γ quickly waned thereafter. iNKT cells from infected animals also became resistant to CD69 upregulation in response to α-GalCer treatment. This refractory period lasted for approximately 1 month. While iNKT cell-deficient mice eliminated BCG as efficiently as wild-type mice, these animals had more granulomas in liver and lung, with signs of caseation, large cellular infiltrates, and some multinucleated macrophages, which were not seen in wild-type animals (106). These findings therefore suggested an anti-inflammatory role for iNKT cells during BCG infection.

### Lymphocytic choriomeningitis virus

Intraperitoneal infection of mice with the Armstrong strain of LCMV caused a selective, long-term loss of iNKT cells in both spleen and liver (93, 107). This apparent loss of iNKT cells was not just due to downregulation of TCR expression. It was observed as soon as 3 days after infection, was most profound around 10 days, and lasted up to 3 months. The dying cells expressed active caspase 3, indicating apoptosis, but this process was independent of Fas/FasL interactions. While the reasons for this sustained loss of iNKT cells remains unclear, it has been suggested to be due to either activation-induced cell death or direct virus infection. Wild-type and CD1d-deficient animals cleared LCMV at similar levels but splenocytes from CD1d-deficient animals produced significantly higher amounts of cytokines (IL-2, IL-4, and IFN-γ), suggesting that iNKT cells suppress the magnitude of the acute antiviral immune response against LCMV (108).

## Interaction of iNKT Cells with the Microbiota

A microbiota is a group of microorganisms that resides in a specific environment. The human host engages in mutualistic relationships with commensal microbes that reside in different parts of the body, especially the gastrointestinal tract. Recent studies have provided evidence that iNKT cells are influenced by the microbiota and that, conversely, iNKT cells can shape the composition of the microbiota (109). Germ-free animals were shown to contain increased numbers of mature, functionally competent iNKT cells in the gut and lung as compared with specific pathogen-free mice (110, 111). These alterations in iNKT cells made germ-free mice more susceptible to tissue damage and inflammation in mouse models of asthma and inflammatory bowel disease. These effects of microbiota on iNKT cells were not limited to mucosal surfaces, as splenic iNKT cells from germ-free animals exhibited reduced expression of activation markers and reduced capacity to produce cytokines. Similar findings were made for fetal human iNKT cells (112), which develop in the absence of microbiota. Fetal small intestinal iNKT cells were phenotypically and functionally mature, whereas their splenic counterparts exhibited reduced expression of activation markers. These findings provide the intriguing possibility to manipulate iNKT cell numbers and functions via the microbiota. The mouse studies have shown that iNKT cells can be reprogrammed in this manner during neonatal but not adult life.

The finding that iNKT cell numbers and functions are influenced by the gut microbiota provides a potential explanation for some of the divergent studies that have been published in the iNKT cell field. For example, pathogenic (113), neutral (114–117), and suppressive (118, 119) roles of iNKT cells in the pathogenesis of obesity-associated inflammation and insulin resistance have been reported (26). This might be caused by differences in the endogenous microbiota in the animal facilities where the different studies were performed.

Mechanisms responsible for the effects of the microbiota on iNKT cell numbers and functions remain unclear but may include recognition of microbe-associated molecular patterns, microbial metabolites, and microbial iNKT cell antigens. With regard to the latter possibility, a provocative study showed that monocolonization of germ-free mice with *B. fragilis* can restore the colonic iNKT cell levels (47). As mentioned above, *B. fragilis* contains α-GalCers that can either activate (43) or inhibit (47) iNKT cells. The inhibitory α-GalCer (Bf717) from this organism was able to limit CD1d-dependent colonic iNKT cell proliferation in germ-free mice (47).

## Summary and Outstanding Questions

The studies discussed here have revealed that iNKT cells become activated during infection by different types of pathogenic as well as commensal microorganisms. While some microorganisms contain iNKT cell ligands, their contribution to iNKT cell activation to the intact organism remains unclear. Innate cytokine-driven pathways play a dominant role in microbial iNKT cell activation. iNKT cells often become activated and produce cytokines early after microbial infection and may transiently expand, contract, or maintain their population size. A common feature of the response of iNKT cells to microbial infection is that it induces long-term unresponsiveness to TCR stimulation.

Despite this progress in our understanding of the response of iNKT cells to microbes, a number of outstanding questions remain to be answered:

- What is the contribution of microbial iNKT cell antigens to the immune response of iNKT cells to microbial pathogens?
- Do endogenous iNKT cell antigens play a role in the response of iNKT cells to microbial pathogens *in vivo*?
- What are the molecular mechanisms responsible for the long-term effects of distinct microbes and their products on the numbers and functions of iNKT cells?
- Can commensal microorganisms or their glycolipid antigens be employed to reprogram iNKT cell functions in humans?
- How do the mechanisms responsible for microbial iNKT cell activation relate to those that mediate iNKT cell activation during sterile inflammation?
- How similar are the mechanisms that control iNKT cell activation in response to microbes in mice and humans, and how do they differ?
- How can this information be employed for the development of improved iNKT cell-based therapies?

These questions will provide rich and fertile avenues for future investigations.

## Conflict of Interest Statement

The authors declare that the research was conducted in the absence of any commercial or financial relationships that could be construed as a potential conflict of interest.
